# Comparative assessment of Alzheimer’s disease-related biomarkers in plasma and neuron-derived extracellular vesicles: a nested case-control study

**DOI:** 10.3389/fmolb.2023.1254834

**Published:** 2023-09-27

**Authors:** Apostolos Manolopoulos, Francheska Delgado-Peraza, Maja Mustapic, Krishna Ananthu Pucha, Carlos Nogueras-Ortiz, Alexander Daskalopoulos, De’Larrian DeAnté Knight, Jeannie-Marie Leoutsakos, Esther S. Oh, Constantine G. Lyketsos, Dimitrios Kapogiannis

**Affiliations:** ^1^ Intramural Research Program, Laboratory of Clinical Investigation, National Institute on Aging, Baltimore, MD, United States; ^2^ Department of Psychiatry and Behavioral Sciences, Johns Hopkins School of Medicine, Baltimore, MD, United States; ^3^ Richman Family Precision Medicine Center of Excellence in Alzheimer’s Disease, Johns Hopkins School of Medicine, Baltimore, MD, United States; ^4^ Division of Geriatric Medicine and Gerontology, Department of Medicine, Johns Hopkins School of Medicine, Baltimore, MD, United States

**Keywords:** extracellular vesicles, biomarkers, precision medicine, neurodegeneration, Alzheimer’s disease

## Abstract

**Introduction:** Alzheimer’s disease (AD) is currently defined according to biomarkers reflecting the core underlying neuropathological processes: Aβ deposition, Tau, and neurodegeneration (ATN). The soluble phase of plasma and plasma neuron-derived extracellular vesicles (NDEVs) are increasingly being investigated as sources of biomarkers. The aim of this study was to examine the comparative biomarker potential of these two biofluids, as well as the association between respective biomarkers.

**Methods:** We retrospectively identified three distinct diagnostic groups of 44 individuals who provided samples at baseline and at a mean of 3.1 years later; 14 were cognitively unimpaired at baseline and remained so (NRM-NRM), 13 had amnestic MCI that progressed to AD dementia (MCI-DEM) and 17 had AD dementia at both timepoints (DEM-DEM). Plasma NDEVs were isolated by immunoaffinity capture targeting the neuronal markers L1CAM, GAP43, and NLGN3. In both plasma and NDEVs, we assessed ATN biomarkers (Aβ_42_, Aβ_40_, total Tau, P181-Tau) alongside several other exploratory markers.

**Results:** The Aβ_42_/Aβ_40_ ratio in plasma and NDEVs was lower in MCI-DEM than NRM-NRM at baseline and its levels in NDEVs decreased over time in all three groups. Similarly, plasma and NDEV-associated Aβ_42_ was lower in MCI-DEM compared to NRM-NRM at baseline and its levels in plasma decreased over time in DEM-DEM. For NDEV-associated proBDNF, compared to NRM-NRM, its levels were lower in MCI-DEM and DEM-DEM at baseline, and they decreased over time in the latter group. No group differences were found for other exploratory markers. NDEV-associated Aβ_42_/Aβ_40_ ratio and proBDNF achieved the highest areas under the curve (AUCs) for discriminating between diagnostic groups, while proBDNF was positively associated with Mini-Mental State Examination (MMSE) score. No associations were found between the two biofluids for any assessed marker.

**Discussion:** The soluble phase of plasma and plasma NDEVs demonstrate distinct biomarker profiles both at a single time point and longitudinally. The lack of association between plasma and NDEV measures indicates that the two types of biofluids demonstrate distinct biomarker signatures that may be attributable to being derived through different biological processes. NDEV-associated proBDNF may be a useful biomarker for AD diagnosis and monitoring.

## 1 Introduction

The 2018 National Institute on Aging—Alzheimer’s Association (NIA-AA) research framework proposed a biomarker-based definition of Alzheimer’s disease (AD) reflecting the three core underlying neuropathological processes: amyloid-β (Aβ) deposition, pathologic tau, and neurodegeneration (ATN) (Jack et al., 2018). To date, positron emission tomography (PET), structural magnetic resonance imaging (MRI), and cerebrospinal fluid (CSF) are the most widely used modalities to derive ATN biomarkers (Hansson, 2021). However, it is recognized that the development and routine use of blood-based biomarkers will improve accessibility, minimize invasiveness and cost, and assist with diagnosis, prediction of disease progression, prognosis, and response to treatments (Angioni et al., 2022).

The soluble blood phase is increasingly being investigated as a potential source of biomarkers. Plasma Aβ_42_/Aβ_40_ may be able to detect abnormal amyloid status in cognitively impaired and cognitively unimpaired individuals even before amyloid PET reaches its positivity threshold (West et al., 2021; Hu et al., 2022). Additionally, several phospho-tau (P-Tau) isoforms can be reliably measured in plasma and demonstrate high precision in predicting Tau deposits in the AD brain (Palmqvist et al., 2020; Mielke et al., 2021). Finally, clinical trials are increasingly utilizing plasma biomarkers either as screening tools (Leuzy et al., 2022) or as surrogates to pharmacodynamic responses (Budd Haeberlein et al., 2022).

Extracellular vesicles (EVs) are membranous nanoparticles that are secreted by all cell-types. Their cargo, encapsulated by a lipid bilayer, comprises nucleic acids, lipids, and proteins that reflect the parental cell identity and homeostatic status (van Niel et al., 2018). EVs secreted by brain cells can be detected in blood, thereby providing a window into the brain, while remaining minimally invasive (Dickens et al., 2017). We and others have isolated neuron-derived EVs (NDEVs) by selective immunoaffinity capture targeting the neuronal cell-adhesion molecule L1CAM and have shown that they can provide predictive, diagnostic, prognostic, and treatment response biomarkers for various brain diseases (Bhargava et al., 2021; Vandendriessche et al., 2022; Blommer et al., 2023). In particular, AD pathogenic proteins (Aβ, Tau, and various P-Tau species) have repeatedly been measured in NDEV preparations and have shown promise for AD clinical and preclinical diagnosis (Fiandaca et al., 2015; Kapogiannis et al., 2019; Eren et al., 2020; Eren et al., 2022). However, studies that simultaneously study biomarkers in the soluble phase of plasma and in plasma NDEVs to assess their comparative biomarker potential are still lacking.

The aim of this study was, primarily, to examine whether core ATN biomarkers (Aβ_42_, Aβ_40_, total Tau, P181-Tau) measured cross-sectionally and longitudinally in both NDEVs and plasma reveal differences in individuals with amnestic MCI (aMCI) who progress to AD dementia compared to individuals with AD dementia or cognitively intact individuals at two time points. Additionally, we assessed the relationship between these NDEV and plasma ATN biomarkers across all individuals and separately within each group. The selection of the groups was based on the hypothesis that individuals with MCI who progress to dementia are at a biologically active disease stage, which may be expressed by dynamic biomarker changes compared to the other two more stable states. Differences between groups were also examined, secondarily, for exploratory biomarkers reflecting additional, and often, overlapping pathologies, synaptic integrity, which weakens early in the course of AD (Clare et al., 2010), and neurotrophic factors, which mediate neuronal survival, maintenance, and regeneration, and have a potentially pathogenetic role in AD with their expression levels being abnormal (Buchman et al., 2016; Lattanzio et al., 2016). These biomarkers included a-synuclein (a-syn), TAR DNA-binding protein 43 (TDP-43), neurogranin (NRGN), P231-Tau, heavy chain neurofilaments (Nf-H), brain-derived neurotrophic factor (BDNF), and precursor brain-derived neurotrophic factor (proBDNF). Specifically, a-syn may be involved in the earliest stages of AD development, reflected in altered CSF levels and associations with brain Aβ plaque deposition in both sporadic and familial AD cases (Twohig et al., 2018; Vergallo et al., 2018). Similarly, NRGN levels are elevated in CSF, depleted in the brain, and associated with poorer cognitive performance in AD patients (Thorsell et al., 2010). Finally, proBDNF and BDNF have been associated with cognitive impairment (Michalski and Fahnestock, 2003; Peng et al., 2005) with several studies showing decreased levels in patients with MCI and AD dementia (Yasutake et al., 2006; Laske et al., 2007) and others showing conflicting results with increased levels in AD patients (Angelucci et al., 2010; Faria et al., 2014).

## 2 Materials and methods

### 2.1 Participants and study design

Forty-four individuals, who had donated blood at two (and two of them at three time points), were identified retrospectively from the biosample bank of the Johns Hopkins Alzheimer’s Disease Research Center (JHADRC). Based on expert consensus panel diagnoses, 14 of these were cognitively unimpaired at all blood collection timepoints (NRM-NRM group); 13 had aMCI at their first blood collection and dementia attributable to AD at later timepoints (MCI-DEM group); and 17 had dementia initially that progressed at later timepoints (DEM-DEM group). The diagnosis of aMCI was established according to the Petersen criteria (Petersen, 2004) and of probable AD according to the revised National Institute of Neurological and Communicative Disorders and Stroke and Alzheimer’s Disease and Related Disorders Association (NINDS-ADRDA) criteria (Dubois et al., 2007). Mean ± standard deviation (SD) time intervals between the first and last blood collection were 3.9 ± 2.4 years for NRM-NRM, 2.9 ± 1.4 years for MCI-DEM, and 2.6 ± 2.4 years for DEM-DEM groups. All blood draws and processing followed established protocols of the JHADRC using standard venipuncture procedures. Preanalytical factors for blood collection and storage comply with guidelines for EV biomarkers (Coumans et al., 2017) as follows: blood samples were collected in EDTA polypropylene tubes, and platelet-free plasma was isolated by, first, sedimenting red blood cells at 2,500 g for 15 min at 4°C, followed by aliquoting the supernatant 0.5 cm above the buffy coat in 0.5 mL fractions stored at −80°C until downstream analysis and NDEV isolation. Hemolysis was ruled out using spectrophotometry (Supplementary Figure S1). All participants provided written informed consent and the study was approved by the Johns Hopkins Institutional Review Board.

### 2.2 NDEV isolation

Plasma aliquots were received and processed blindly by investigators at the National Institute on Aging, Baltimore, MD. NDEVs were isolated following a two-step procedure based on previous studies by our group and others validating L1 Cell Adhesion Molecule (L1CAM), the axonal protein Growth-Associated Protein 43 (GAP43), and the neuron cell surface protein Neuroligin 3 (NLGN3) as targets for the immunocapture of NDEVs from plasma (Kapogiannis et al., 2019; Eitan et al., 2023). Slight differences between the protocol employed here and that described by Eitan et al. include the isolation of crude plasma EVs via size exclusion chromatography (SEC) instead of polymer-based sedimentation, and targeting the neuronal surface proteins GAP43 and NLGN3, in addition to L1CAM, for the immunocapture of NDEVs, expected to increase the purity and yield of recovered NDEVs, respectively. Modifications made did not affect the enrichment of NDEVs as shown by EV characterization experiments (Supplementary Figures S3, S4).

Initially, 250 μL of plasma were loaded into a SmartSEC™ HT plate stacked over a collection plate (System Biosciences LCC, Palo Alto, CA). After incubation for 30 min at room temperature (RT) and centrifugation at 500 g for 1 min, the first fraction of purified EVs was collected into the collection plate. Next, an equal volume of SmartSEC™ Isolation buffer was added, followed by centrifugation at 500 g for 1 min, generating the second EV fraction. The two fractions of purified total EVs were combined into a sterile 2 mL microtube and were incubated for 2 h at RT with gentle rotation after the addition of 5 μg of biotinylated anti-human L1CAM antibody (clone 5G3, eBioscience, San Diego, CA) and 10 μg of GAP43 and NLGN3 ExoSORT antibody mix (NeuroDex, Natick, MA). The EV-antibody complexes were then incubated with 75 μL of washed Dynabeads™ MyOne™ Streptavidin T1 (Invitrogen, Thermo Fisher Scientific Inc., Waltham, MA) for 1 h at RT with rotation. The NDEV-bead complexes were collected using a magnetic separator and transferred into ExoSORT elution buffer. The elution of NDEVs was performed for 5 min at 50°C, followed by bead separation and transfer of the eluate into a clean microtube containing Tris-HCl (1M, pH 8.0) buffer (Invitrogen, Thermo Fisher Scientific Inc., Waltham, MA). The NDEV suspension was subjected to EV lysis by adding RIPA lysis buffer (MilliporeSigma Corp., Burlington, MA) (containing 0.08% SDS) supplemented with 1X protease/phosphatase inhibitors, and the resulting solution was stored at −80°C until downstream immunoassays.

### 2.3 Plasma and NDEV protein quantification

In both plasma and NDEV lysates, we used the single molecule array (SimoaⓇ) technology to measure levels of Aβ_42_, Aβ_40_, total Tau and P181-Tau (Quanterix Corp., Billerica, MA). Additionally, in NDEV lysates we quantified several exploratory markers, including a-syn, TDP-43, NRGN, P231-Tau, Nf-H, BDNF, utilizing an Exosome Characterization 6-Plex Human ProcartaPlex™ Simplex™ Panel (Invitrogen, Thermo Fisher Scientific Inc., Waltham, MA), and proBDNF by ELISA (Biosensis Pty Ltd, Thebarton, Australia) using the Synergy™ H1 microplate reader set to 450 nm and the Gen5™ microplate data collection software (BioTek Instruments Inc., Winooski, VT). SDS concentration contained in initial NDEV lysates decreased up to 0.04% depending on the assay. Plates were read using the Luminex^®^ 100/200TM instrument with the xPOTENT^®^ acquisition software (Luminex Corp., Austin, TX). All samples of a given participant were included on the same plate to avoid within-subject variability caused by inter-plate variability. In all assays, samples were assessed in duplicate.

### 2.4 NDEV characterization

For the validation of EV identity of isolated particles, we used a Human ProcartaPlex™ Simplex™ assay to comparatively quantify levels of CD9, CD63, and CD81 in NDEVs, pan-tetraspanin-EVs (panTET-EVs), total EVs, neat plasma, and beads only (sample where antibody was omitted in immunoprecipitation step of the isolation protocol that should reflect non-specific binding). Additionally, levels of tetraspanins CD9, CD63 and CD81, along with Cytochrome C, Syntenin-1, and Very Late Antigen-4 (VLA-4, also known as integrin α4β1) were quantified utilizing an in-house customized panel. The assessment of Syntenin-1 and VLA-4 as EV markers was based on recent proteomic studies in EVs, derived from multiple cell lines and brain tissue, that qualified them as canonical EV markers along with CD9, CD63, and CD81 (Kugeratski et al., 2021; You et al., 2022). Moreover, Cytochrome C, a mitochondrial protein, was formerly used as a negative control to assess the co-isolation of EVs with cellular components, however, more recently, it has also been shown to be actively sorted into some EVs (Todkar et al., 2021). The diameter and concentration of intact EVs was determined using nanoparticle tracking analysis (NTA) (Nanosight NS500 equipped with a 532 nm laser module; Malvern, Amesbury, United Kingdom), based on the Brownian motion of particles detected by light scattering. 10 μL of intact NDEVs were diluted in PBS-1X until detecting 20–100 particles per frame, an optimal concentration range that minimizes detection of EVs under the size limit resolution of the instrument (50–100 nm) and overestimation of EV size due to particle saturation. Values for particles per mL and diameter mean and mode were averaged from 5 videos of 20 s each. To visually confirm the EV identity of isolated particles, we used transmission electron microscopy (TEM) and a NanoView^®^ pan-tetraspanins fluorescent assay (Nanoview Biosciences, Brighton, MA) that captures five different probes [i.e., CD9, CD41, CD63, CD81, and mouse IgG (MIgG)] using detection probes for CD9, CD63, and CD81 on ExoView™ chip. Finally, neuronal origin of isolated EVs was assessed with Western blotting, probing for the neuronal markers Neuronal nuclear protein (NeuN), Glutamate ionotropic receptor AMPA type subunit 2 (GluR2), and Enolase 2 (ENO2). NDEVs were compared to total EVs, beads only, and brain lysate as a positive control.

### 2.5 Statistical analysis

We compared participant characteristics by group using either ANOVA for continuous variables or Pearson’s χ^2^ tests for categorical variables. We used Box-Cox transformed values in regression models due to the non-normal distributions of many of the NDEV and plasma markers (Sakia, 1992). Baseline comparisons between groups were made using linear regression models adjusting for participant age, sample age, and sex with the NRM-NRM group as the reference category and dichotomous indicator variables denoting MCI-DEM or DEM-DEM. To compare change in markers over time between groups, we fitted linear mixed effects models with random intercepts similar to the baseline comparison models but with terms for time and group by time interactions. From these models, fitted change over time by group could be calculated for each marker, and compared between groups. Associations between NDEV and plasma markers and between both types of markers and Mini-Mental State Examination (MMSE) scores were assessed by fitting longitudinal mixed effects models with random intercepts, adjusting for participant age, sample age, and sex. These models did not include a term for time, therefore the interpretation of the regression coefficient is the association between the marker and MMSE score measured at the same time point, with this effect being assumed invariant across time points. To determine the utility of each of the markers in predicting group membership, we calculated Somer’s D and area under the curve (AUC) (Newson, 2002). All hypothesis tests were two-sided; due to the exploratory nature of our work, we did not adjust *p*-values for multiple comparisons.

## 3 Results

### 3.1 Participant characteristics

Mean ± SD age of participants at first blood collection was 77.1 ± 7.4, 72.0 ± 8.3, and 77.4 ± 6.6 years old for NRM-NRM, MCI-DEM, and DEM-DEM groups, respectively. All groups had similar composition of males and females. Results of MMSE testing confirmed more profound cognitive losses at baseline for DEM-DEM group than the other two groups. Study cohort baseline characteristics are provided in Table 1.

**TABLE 1:** Participant baseline characteristics.

Characteristic	NRM-NRM (*N* = 14)	MCI-DEM (*N* = 13)	DEM-DEM (*N* = 17)	
Age—years, mean (SD)	77.1 (7.4)	72.0 (8.3)	77.4 (6.6)	*p* = 0.11
Sex—no. (%)				*p* = 0.16
Male	4 (29.0)	4 (31.0)	10 (59.0)	
Female	10 (71.0)	9 (69.0)	7 (41.0)	
Race—no. (%)				*p* = 0.27
White/Caucasian	12 (86.0)	9 (69.0)	15 (88.0)	
Black/African American	1 (7.0)	4 (31.0)	2 (12.0)	
Asian	1 (7.0)	0 (0.0)	0 (0.0)	
MMSE—mean (SD)	29.7 (0.5)	27.6 (2.4)	25.1 (3.4)	*p* < 0.001
APOE ε4 allele—no. (%)				*p* = 0.003
0	11 (79.0)	2 (15.0)	6 (38.0)	
1 or 2	3 (21.0)	11 (85.0)	10 (63.0)	
Length of follow-up—years, mean (SD)	3.9 (2.4)	2.9 (1.4)	2.6 (2.4)	*p* = 0.27

APOE, Apolipoprotein E; MMSE, Mini-Mental State Examination. *p* values are derived from ANOVA tests for continuous variables and Pearson’s χ^2^ tests for categorical variables.

### 3.2 NDEV characterization

The comparative assessment of CD9, CD63, and CD81 levels in NDEVs, panTET-EVs, total EVs, neat plasma, and beads only showed enrichment of canonical EV markers in NDEVs and panTET-EVs, with levels of CD63 being significantly lower compared to those of CD9 and CD81 (Supplementary Figure S4B). Additionally, quantification of tetraspanin markers and Cytochrome C, Syntenin-1, and VLA-4 confirmed the EV identity of isolated particles with their levels at baseline being similar among the three diagnostic groups (Supplementary Figures S2C–H). NTA showed a diameter range of particles of 144–230 nm typical of plasma EVs with their average diameter at baseline being similar among groups (Supplementary Figures S2B, S3A); however, the NDEV concentration of the DEM-DEM group at baseline was significantly lower compared to that of the NRM-NRM group (Supplementary Figure S2A). Moreover, the morphology and size of particles assessed by TEM were compatible with those of EVs (Supplementary Figure S3B), while the absence of CD41, as a platelet marker, in NanoView^®^ fluorescent assay (Supplementary Figures S3C, D), further validated the purity and EV identity of isolated particles. Finally, Western blotting revealed enrichment of neuronal markers in NDEVs compared to total EVs and beads only confirming the neuronal origin of EVs (Supplementary Figure S4A).

### 3.3 NDEV-associated and plasma markers at baseline

**FIGURE 1:**
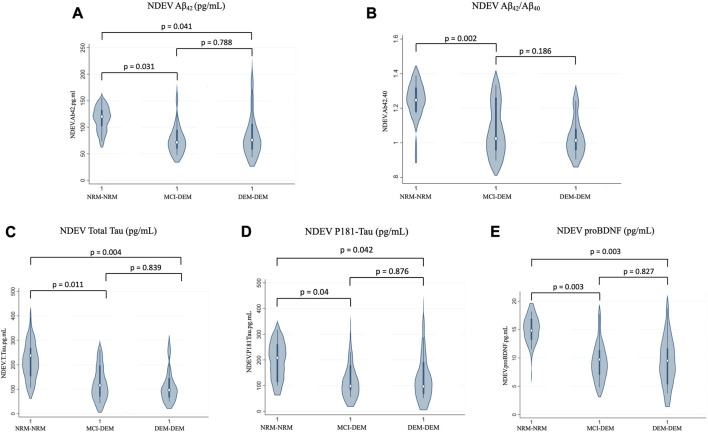
Baseline differences of NDEV markers between groups. **(A)** Aβ_42_ (pg/mL); **(B)** Aβ_42_/Aβ_40_; **(C)** Total Tau (pg/mL); **(D)** P181-Tau (pg/mL); **(E)** proBDNF (pg/mL). 1 = Visit 1.

Compared to NRM-NRM, both MCI-DEM and DEM-DEM groups had lower Box-Cox transformed values of NDEV-associated Aβ_42_ [Beta ± standard error (SE) −0.11 ± 0.05, *p* = 0.03 and −0.10 ± 0.05, *p* = 0.04, respectively] (Figure 1A). No difference between groups was found for Aβ_40_; however, the Aβ_42_/Aβ_40_ ratio was decreased in MCI-DEM compared to NRM-NRM (−0.13 ± 0.04, *p* = 0.002) (Figure 1B). Of note, total Tau and P-181 Tau were also decreased in NDEVs of MCI-DEM (−0.40 ± 0.15, *p* = 0.01 and −0.14 ± 0.07, *p* = 0.04, respectively) and DEM-DEM (−0.43 ± 0.14, *p* = 0.004 and −0.13 ± 0.06, *p* = 0.04, respectively) compared to NRM-NRM group (Figures 1C, D). Finally, regarding the exploratory NDEV-associated markers, differences were only observed for proBDNF, which was lower in both MCI-DEM (−7.16 ± 2.27, *p* = 0.003) and DEM-DEM (−6.67 ± 2.12, *p* = 0.003) groups compared to NRM-NRM (Figure 1E).

**FIGURE 2:**
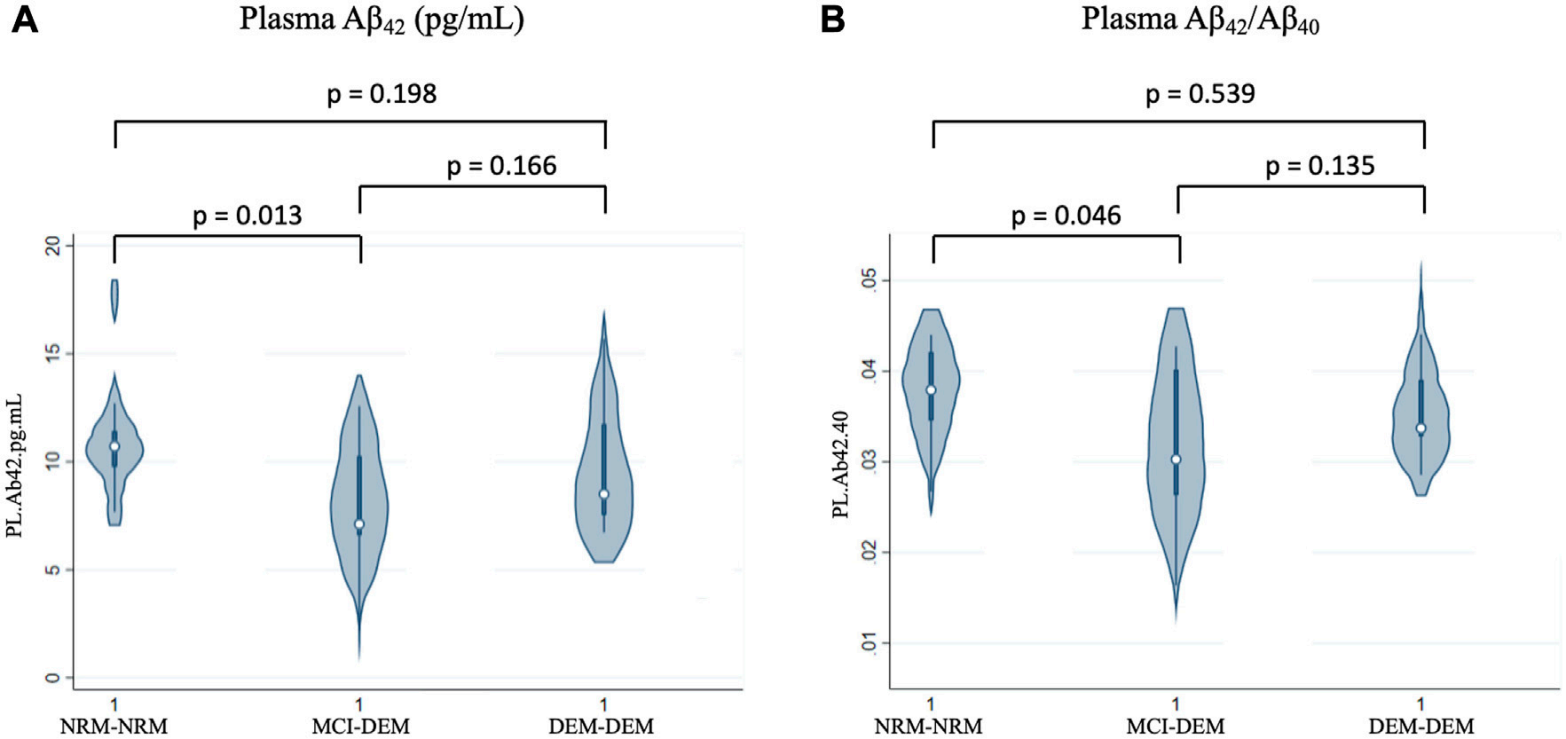
Baseline differences of plasma markers between groups. **(A)** Aβ_42_ (pg/mL); **(B)** Aβ_42_/Aβ_40_; 1 = Visit 1.

In plasma, similarly to NDEVs, Aβ_42_ and Aβ_42_/Aβ_40_ were lower in MCI-DEM compared to NRM-NRM group (−1.80 ± 0.69, *p* = 0.01 and −0.001 ± 0.00, *p* = 0.05) (Figures 2A, B). However, no group differences were observed for Aβ_40_ or Tau. Of note, for both plasma and NDEVs, MCI-DEM and DEM-DEM groups did not differ from each other on any assessed marker.

### 3.4 NDEV-associated and plasma marker longitudinal changes

**FIGURE 3:**
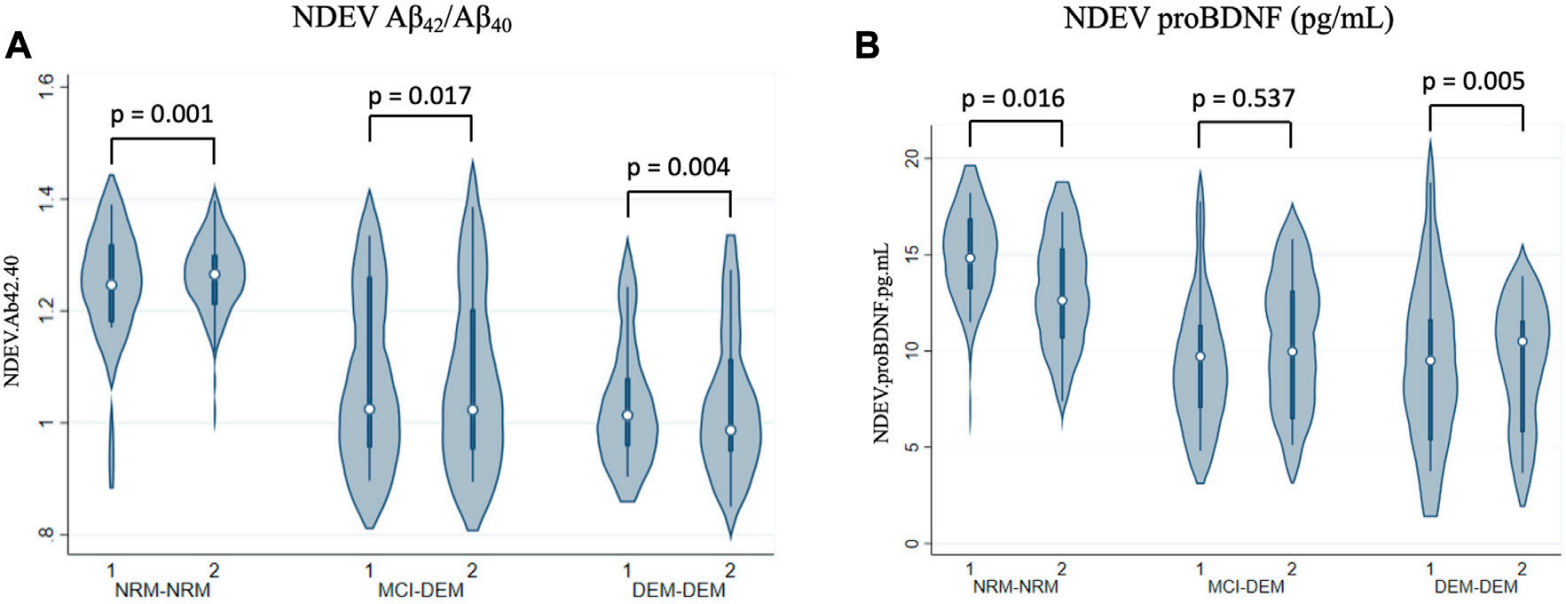
Longitudinal changes of NDEV markers. **(A)** Aβ_42_/Aβ_40_; **(B)** proBDNF (pg/mL). 1 = Visit 1; 2 = Visit 2.

NDEV-associated Aβ_42_/Aβ_40_ decreased over time in all three diagnostic groups, at a similar rate of change per year across all three (Box-Cox transformed values: −0.02 ± 0.01, *p* = 0.001 for NRM-NRM, −0.02 ± 0.01, *p* = 0.02 for MCI-DEM, and −0.01 ± 0.01, *p* = 0.004 for DEM-DEM group) (Figure 3A). Additionally, NDEV-associated proBDNF decreased over time in DEM-DEM (−0.94 ± 0.33, *p* = 0.01) and NRM-NRM (−0.70 ± 0.29, *p* = 0.02) groups at a similar rate (Figure 3B). Finally, NDEV-associated Aβ_42_, Aβ_40_, Tau biomarkers and other exploratory markers did not show any longitudinal changes in any group.

**FIGURE 4:**
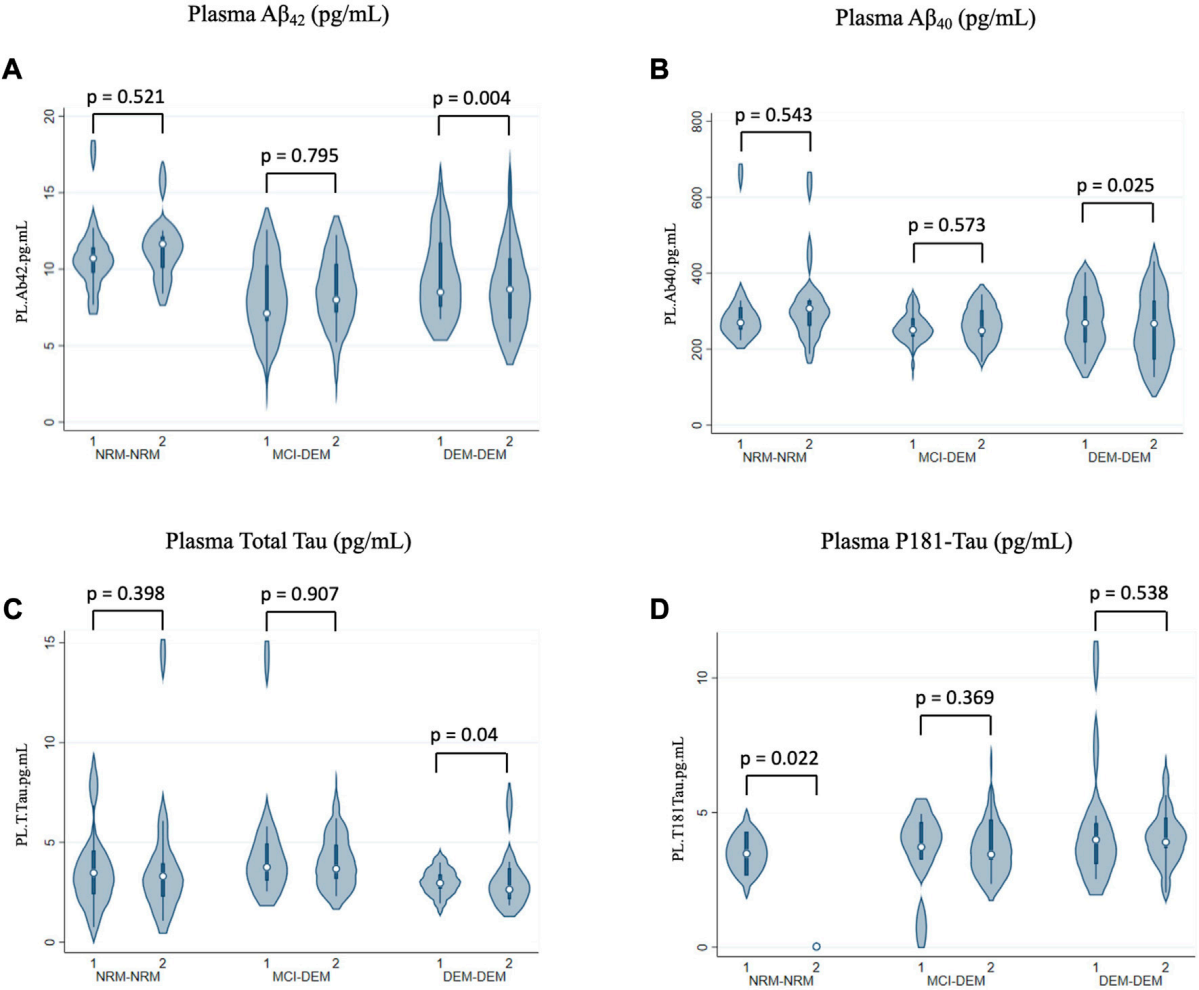
Longitudinal changes of plasma markers. **(A)** Aβ_42_ (pg/mL); **(B)** Aβ_40_ (pg/mL); **(C)** Total Tau (pg/mL); **(D)** P181-Tau (pg/mL). 1 = Visit 1; 2 = Visit 2.

In plasma, levels of Aβ_42_ and Aβ_40_ decreased over time in DEM-DEM (−0.28 ± 0.10, *p* = 0.004 and −0.03 ± 0.01, *p* = 0.03, respectively), but not in any other group (Figures 4A, B). Conversely, total Tau levels increased over time in DEM-DEM (0.06 ± 0.03, *p* = 0.04) (Figure 4C). Finally, NRM-NRM showed a decrease over time in P181-Tau levels (−2.32 ± 1.01, *p* = 0.02) unlike the other two groups (0.07 ± 0.08, *p* = 0.37 for MCI-DEM and 0.04 ± 0.06, *p* = 0.54 for DEM-DEM) (Figure 4D). Non-Box-Cox-transformed values of biomarkers at each time point are provided in Supplementary Table S1.

### 3.5 Associations and ROC analysis

**FIGURE 5:**
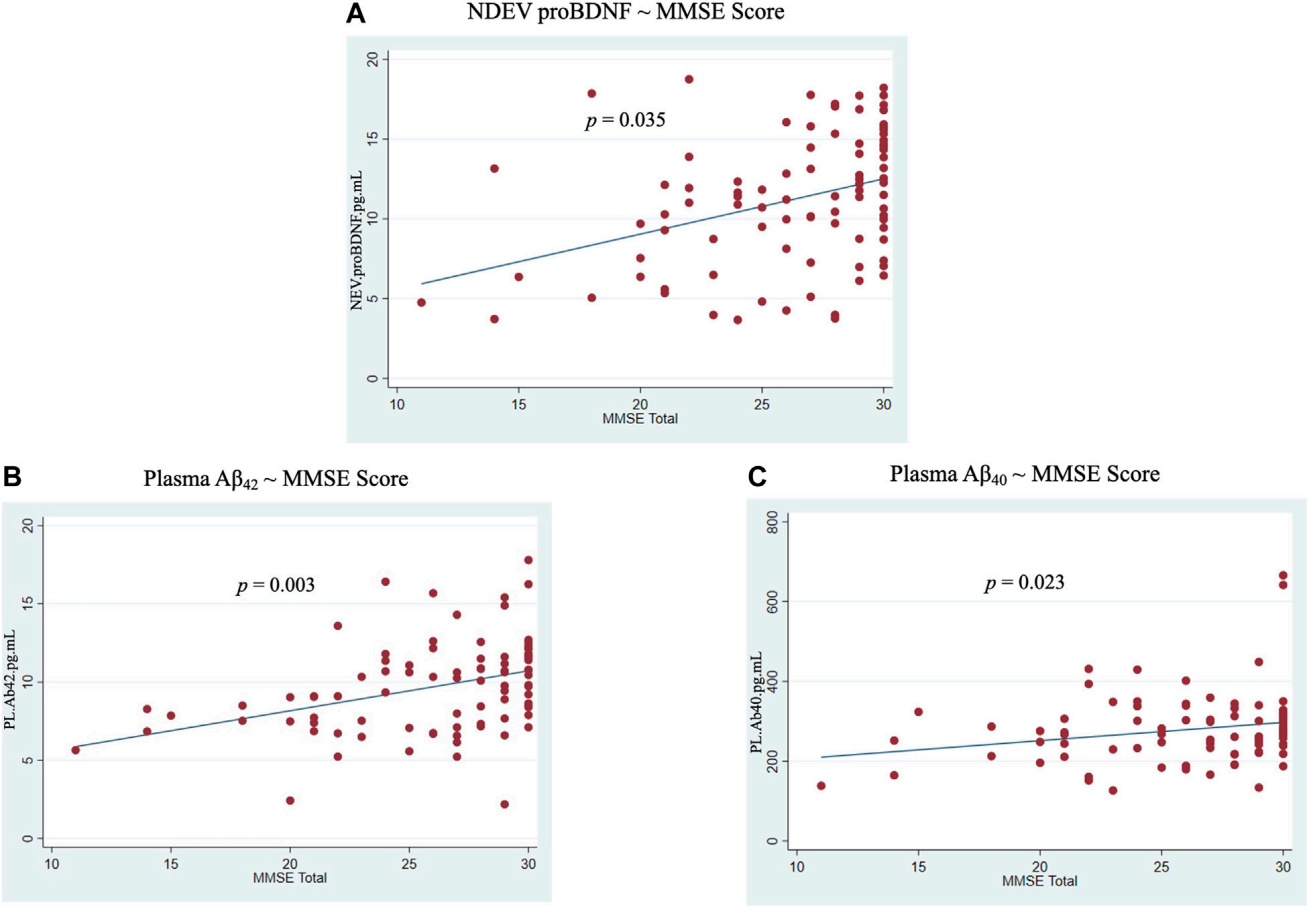
Associations between markers and MMSE score across all participants. **(A)** NDEV proBDNF ∼ MMSE score; **(B)** Plasma Aβ_42_ ∼ MMSE score; **(C)** Plasma Aβ_40_ ∼ MMSE score.

No significant associations were revealed between NDEVs and plasma for any ATN biomarker across or within diagnostic groups (Supplementary Figure S7). However, NDEV-associated proBDNF was positively associated with MMSE score across the entire cohort (0.17 ± 0.08, *p* = 0.04) (Figure 5A). In plasma, positive associations with MMSE score were observed for Aβ_42_ and Aβ_40_ (0.76 ± 0.24, *p* = 0.003 and 5.33 ± 2.30, *p* = 0.02, respectively) (Figures 5B, C). Additionally, ROC analysis showed that of all NDEV and plasma markers, NDEV-associated Aβ_42_/Aβ_40_ achieved the highest AUC (Somers’ D = −0.48, AUC = 74.2%) in discriminating between diagnostic groups followed by NDEV-associated proBDNF (Somers’ D = −0.44, AUC = 72.1%).

## 4 Discussion

In this study, we utilized a cohort of three distinct and well-characterized diagnostic groups and measured longitudinally the core ATN biomarkers, in both plasma and NDEVs, along with several other exploratory markers reflecting additional pathologies, synaptic integrity, and neurotrophic factors. Unlike past studies, NDEVs were isolated from plasma samples by immunoaffinity capture targeting three separate neuronal markers, and not solely L1CAM, to yield a wider range of neuronal origin EVs. NDEV-based measures revealed group differences at baseline even when plasma did not, while the two types of biofluids did not show any association for any assessed marker. The Aβ_42_/Aβ_40_ ratio measured either in plasma or NDEVs was lower in MCI-DEM than NRM-NRM group at baseline, whereas NDEV-associated proBDNF was lower in MCI-DEM and DEM-DEM groups compared to NRM-NRM, with its levels decreasing longitudinally as dementia was advancing. Interestingly, these two biomarkers achieved the highest AUCs for discriminating between diagnostic groups, while additionally, NDEV-associated proBDNF was positively correlated with MMSE score. Paradoxical findings emerged for NDEV-associated total Tau and P181-Tau, with the direction of differences at baseline being the opposite from what had previously been observed in other cohorts (Fiandaca et al., 2015; Kapogiannis et al., 2019; Eren et al., 2020). Besides proBDNF, no group differences were found for the rest of exploratory markers.

Additionally, we performed a comprehensive assessment of the EV composition of each sample by measuring particle composition by NTA, as well as measurement of CD9, CD63, and CD81 (tetraspanin transmembrane EV markers) as well as Syntenin-1, VLA-4, and Cytochrome C (intravesicular EV markers reflecting different pathways of biogenesis) by ProcartaPlex™ Simplex™ multiplex EV assay. If there were any systematic differences in EV composition between groups, one would expect this to be reflected in multiple measures. This was not the case, and the only group difference observed was for NDEV concentration by NTA, which was decreased in the DEM-DEM group at baseline compared to the other two groups. One should bear in mind that neither NTA nor any of the bead-based EV assays like the ones employed in this study, can detect total EVs originating from all EV biogenesis pathways. For example, NTA has a size limit resolution of approximately 50 nm, at best, which is influenced by the instrument’s light scattering detection sensitivity (practically, a large portion of EVs < 100 nm goes unobserved). Thus, NTA EV concentration reflects primarily larger EVs and excludes a large portion of smaller EVs mainly of endosomal origin (where many biomarkers used in this study originate). On the other hand, bead assays based on the capture and detection of pan-tetraspanins as canonical EV markers might exclude other subpopulations of small EVs, presumably originating from both the endosomal pathway as well as the plasma membrane, previously reported to be devoid of CD9/CD63/CD81 (Kowal et al., 2016). Therefore, the discrepancies in the NDEV concentration between diagnostic groups obtained from NTA and the multiplex EV assays render the observed difference for the DEM-DEM group at a single time point questionable.

The assessment of tetraspanin markers, along with Cytochrome C, Syntenin-1, and VLA-4 showed that NDEVs carried increased levels of CD9, CD81 and Syntenin-1, compared to CD63 and VLA-4 (Supplementary Figures S2C–H, S4B). These results are in line with proteomics studies (Pulliam et al., 2019; Anastasi et al., 2021) and the NanoView^®^ pan-tetraspanins fluorescent assay (Supplementary Figures S3C, D) showing low to undetectable CD63 levels, comparable to those of Cytochrome C. Additionally, diameter range of particles was 144–230 nm as determined by NTA, similar to those from JHADRC and other cohorts (Kapogiannis et al., 2019; Eren et al., 2022); however, others have shown deviating average values (e.g., 100–125 nm) (Nogueras-Ortiz et al., 2020). Aside from expected size differences due to subject-to-subject variability across cohorts, size determination via NTA is subject to considerable technical variability originating from fluctuating sample dilutions, which in turn are required for optimal image processing. As previously reported (Bachurski et al., 2019) and validated by our group, fold dilution of plasma derived EVs is negatively correlated with NTA particle size mode (Supplementary Figures S5D, S6H). Although this source of technical variability might result in inaccurate size determination across studies, our objective of comparing EV size differences across the experimental groups of the present study was met by measuring samples at an optimal particle density range achieving reduced size variability (Supplementary Figure S5D).

Regarding the Aβ_42_/Aβ_40_ ratio measured in plasma, our findings are in line with those of other studies that examined plasma amyloid levels in longitudinal cohorts. Perez-Grijalba et al. (2019) reported lower Aβ_42_/Aβ_40_ levels in MCI subjects compared to controls, which were also negatively correlated with cerebral amyloid deposition and predicted future development of dementia. Similarly, in another study, low plasma Aβ_42_/Aβ_40_ levels were associated with more pronounced cognitive decline over time (Giudici et al., 2020). Additionally, in our study, the numerator of the ratio, Aβ_42_, measured in plasma was also lower in MCI-DEM compared to NRM-NRM group at baseline, decreased over time in DEM-DEM group, and was positively correlated with MMSE score. Similarly, in NDEVs, its levels were lower in MCI-DEM and DEM-DEM groups compared to NRM-NRM. Although the latter finding comes in contrast with some other studies of NDEVs (Fiandaca et al., 2015; Jia et al., 2019), in a study of pathologically confirmed AD cases from the same site (JHADRC), lower rather than higher NDEV-associated Aβ_42_ levels were associated with worse cognitive performance (Eren et al., 2022). In the largest longitudinal study of blood NDEV biomarkers for AD prediction, no differences were seen in Aβ_42_ between individuals who would go on to develop AD compared to those who would remain cognitively normal (Kapogiannis et al., 2019). These conflicting results may be attributable to the fact that Aβ is associated with the external surface of plasma EVs rather than true intra-vesicular cargo (Eitan et al., 2016). Operationally, they raise doubts as to whether NDEV-associated Aβ_42_ is a reliable biomarker for disease diagnosis or monitoring.

NDEV-associated proBDNF produced significant results in multiple analyses, including lower levels in MCI and AD dementia compared to the cognitively normal state and further declines with dementia progression, a positive correlation with MMSE score, and the second best performance in group classification by ROC analysis. Unlike proBDNF, mature BDNF showed no differences between groups. These findings are in agreement with the study by Eitan et al. (2023), in which levels of NDEV-associated proBDNF were also found lower in AD compared to control individuals and were positively correlated with MMSE scores. Our results, also, indicate that levels of NDEV-associated proBDNF may reflect true relative concentrations in brain cells, since similar decreases in MCI and AD dementia compared to the cognitively normal state were found in an autopsy study using cerebral tissue samples (Peng et al., 2005). Moreover, NDEV-associated proBDNF has shown biomarker properties for additional age-related conditions. In a longitudinal study using samples from the Baltimore Longitudinal Study of Aging, increased levels of proBDNF in L1CAM (+) NDEVs were associated with walking speed decline (Suire et al., 2017). Overall, these results indicate that NDEV-associated proBDNF may be a useful biomarker for diagnosis and disease monitoring.

The inclusion of well-characterized subjects, the availability of longitudinal measurements spanning a mean of 3.1 years and the fact that both plasma and plasma NDEVs were assessed simultaneously are considerable strengths of this study. Moreover, this is the first study using a combination of antibodies against L1CAM, GAP43, and NLGN3 to derive NDEVs. Nevertheless, important limitations must be recognized. First, even though we utilized three well-characterized diagnostic groups, we did not study MCI individuals who remained stable over time as a potentially interesting contrast to those with MCI who progressed to dementia. Additionally, a paradoxical result emerged for NDEV-associated total Tau and P181-Tau biomarkers, with the differences observed at baseline being the opposite from what had previously been observed. On the other hand, no differences were found between groups for P231-Tau, contradicting previously reported strong correlations between the two phosphorylated forms (Bayoumy et al., 2021). Although, this may be attributable to the new type of NDEVs we isolated, it may also represent a peculiarity of this small cohort. Additionally, in this study, we measured P181-Tau levels using the SimoaⓇ technology. Nevertheless, in a study by Mielke et al. (2021) that comparatively assessed various analytical assays, P181-Tau levels were correlated with cognitive performance only when measured with the Meso Scale Discovery platform, and not with SimoaⓇ. Although, in that study, P181-Tau was assessed in the soluble phase of plasma, these findings regarding the analytical assays performance may also have implications and extend to the study of EVs. Moreover, NDEV-associated total Tau has shown a lack of association with cognitive performance (Eren et al., 2022), questioning its suitability as a disease monitoring biomarker. Collectively, these observations warrant further research on NDEVs isolated by immunoaffinity capture targeting the combination of L1CAM, GAP43, and NLGN3, using various analytical assays for the assessment of Tau biomarkers.

Importantly, our study generated a novel hypothesis to inform future research. The complete lack of association between NDEVs and plasma for any assessed marker may indicate that the two types of biofluids demonstrate distinct biochemical signatures, reflecting the intra- and extracellular environment, respectively. Additionally, although Tau is highly abundant in the central nervous system (CNS), it is also present in peripheral tissues (e.g., liver, kidney, heart) implying that Tau forms measured in plasma originate, to some extent, from non-CNS sources (Dugger et al., 2016). In line with this argument, Barthelemy et al. estimated that only a fifth of the signal measured by plasma total Tau is brain-derived while the remainder originates from peripheral sources (Barthelemy et al., 2020). Similarly, Aβ peptides are generated in appreciable amounts from sources outside CSN (e.g., skeletal muscles, platelets, vascular wall) allowing for an active and dynamic interchange between the brain and periphery (Li et al., 1998; Kuo et al., 2000; Van Nostrand and Melchor, 2001). Although, this lack of association between the two biofluids may be attributable to the different nature of their biomarkers, it also assuages concerns that NDEV markers of neurodegenerative diseases simply represent soluble contaminants or non-specific association with the EV protein corona. Multiple lines of evidence implicate EVs in AD pathogenesis: intracellularly generated Aβ may be released into the extracellular compartment associated with EVs, facilitating its uptake by recipient cells causing cytotoxicity (Toh and Gleeson, 2016; Sardar Sinha et al., 2018). Similarly, for Tau, convincing evidence exists that EVs act as vehicles for its trans-synaptic spreading (Wang et al., 2017). Our findings suggest that EV Aβ and Tau cargos may vary dramatically depending on the stage of disease, with levels varying over time, something that ought to be seriously considered when attempting to address their pathogenic significance. Moreover, our findings regarding NDEV-associated proBDNF encourage the conduct of further longitudinal cohort studies that could validate its potential as a diagnostic and disease monitoring biomarker, but also examine its prognostic and predictive value.

## Data Availability

The raw data supporting the conclusion of this article will be made available by the authors, without undue reservation.
