# Supplementary material for: Comparative assessment of Alzheimer’s disease-related biomarkers in plasma and neuron-derived extracellular vesicles: a nested case-control study
Source: Front Mol Biosci. 2023 Sep 27;10:1254834. doi: 10.3389/fmolb.2023.1254834 (PMC10565036; doi:10.3389/fmolb.2023.1254834)
Supplement: Supplementary file 1 [file DataSheet1.pdf]

Supplementary Material

Comparative Assessment of Alzheimer's Disease-Related Biomarkers in Plasma and Neuron-Derived Extracellular Vesicles: a Nested Case-Control Study

Apostolos Manolopoulos[1*], Francheska Delgado-Peraza[1], Maja Mustapic[1], Krishna Ananthu Pucha[1], Carlos Nogueras-Ortiz[1], Alexander Daskalopoulos[1], De'Larrian DeAnté Knight[1], Jeannie-Marie Leoutsakos[2,3], Esther S Oh[3,4], Constantine G Lyketsos[2,3], Dimitrios Kapogiannis[1*]

[1]Intramural Research Program, Laboratory of Clinical Investigation, National Institute on Aging, Baltimore, MD, USA.

[2]Department of Psychiatry and Behavioral Sciences, Johns Hopkins School of Medicine, Baltimore, MD, USA.

[3]Richman Family Precision Medicine Center of Excellence in Alzheimer's Disease, Johns Hopkins School of Medicine, Baltimore, MD, USA.

[4]Department of Medicine, Division of Geriatric Medicine and Gerontology, Johns Hopkins School of Medicine, Baltimore, MD, USA.

*** Correspondence:**
Apostolos Manolopoulos, M.D., M.Sc.
apostolos.manolopoulos@nih.gov

Dimitrios Kapogiannis, M.D.
kapogiannisd@mail.nih.gov

Figure legends

Figure S1. Assessment of hemolysis using a previously described method (Harboe, 1959). Series of gradually hemolyzed standards were prepared by diluting a completely hemolyzed sample (100%) with non-hemolyzed plasma (0%). (**A**) Absorbance of Hemoglobin at 415 nm in standards; (**B**) Hemoglobin absorbance of standards and 20 plasma samples isolated using the methodology described in the manuscript; (**C**) Visual representation of results.

Figure S2. NDEV Characterization. Comparison of (**A**) NDEV Concentration (n/mL); (**B**) NDEV mean diameter (nm); (**C**) NDEV CD9 (pg/mL); (**D**) NDEV CD63 (pg/mL); (**E**) NDEV CD81 (pg/mL); (**F**) NDEV Cytochrome C (pg/mL); (**G**) NDEV Syntenin-1 (pg/mL); (**H**) NDEV Very Late Antigen-4 (VLA-4) (pg/mL) between diagnostic groups. 1 = Visit 1; 2 = Visit 2; ns = not significant.

Figure S3. NDEV Characterization. (**A**) Graphical representation of NDEV size distribution and concentration determined by nanoparticle tracking analysis (NTA); (**B**) Transmission electron microscopy (TEM) image (negative staining, scale 200 nm) of isolated particles fitting the profile of EVs; (**C**) NanoView® pan-tetraspanins fluorescent assay showing fluorescently labeled detection probes (CD9, CD63, CD81) on ExoView™ chip; (**D**) Graphical representation of EVs captured on a chip with five different probes (CD9, CD41, CD63, CD81, and MIgG). Absence of CD41 as a platelet marker reflects the purity of EV isolation. CD9, CD63, and CD81 are canonical markers and MIgG shows a level of nonspecific binding. MIgG = Mouse IgG.

Figure S4. NDEV Characterization. (**A**) Western blot, probing for the neuronal markers NeuN, GluR2, and ENO2, that shows neuronal enrichment in NDEVs, compared to Total EVs, and beads only; (**B**) Validation of the isolation method, using a Human ProcartaPlex™ Simplex™ assay, that shows enrichment of canonical EV markers in NDEVs and pan-tetraspanin-EVs, compared to Total EVs, neat plasma, and beads only. TEV = Total EVs; BO = beads only; NeuN = Neuronal nuclear protein; GluR2 = Glutamate ionotropic receptor AMPA type subunit 2; ENO2 = Enolase 2; panTET-EVs = pan-tetraspanin-EVs.

Figure S5. Determination of optimal particle per frame (PPF) range using 200 nm beads. (**A**) PPF in function of sample fold dilution; (**B**) PPF range showing a linear relationship with sample dilution; (**C**) Size distribution of beads at different concentrations/PPF; (**D**) Assessment of the effect of sample concentration (PPF) on particle size mode.

Figure S6. Validation of optimal particle per frame (PPF) range using crude total EVs isolated from human plasma. (**A**, **C**, **E**) PPF in function of sample fold dilution; (**B**, **D**, **F**) PPF range showing linear relationship with sample dilution in crude EVs from the plasma of three different subjects; (**G**) Mean size distribution of crude EVs from the plasma of one human subject analyzed at concentrations providing PPFs either within the optimal range resulting in a linear relationship with sample dilution (red line), or at lower PPFs (black line). EVs/mL values have been corrected for dilution factor; (**H**) Min-to-max particle size mode distribution of crude EVs from the plasma of three human subjects analyzed at PPFs under (low PPF), within (optimal PPF), or above (high PPF) the optimal range. Statistical analysis: two-way ANOVA.

Figure S7. Associations between NDEV and plasma markers within each diagnostic group. (**A**) NDEV Aβ_{42} ~ Plasma Aβ_{42}; (**B**) NDEV Aβ_{40} ~ Plasma Aβ_{40}; (**C**) NDEV Aβ_{42}/Aβ_{40} ~ Plasma Aβ_{42}/Aβ_{40}; (**D**) NDEV Total Tau ~ Plasma Total Tau; (**E**) NDEV P181-Tau ~ Plasma P181-Tau.

Figure S1

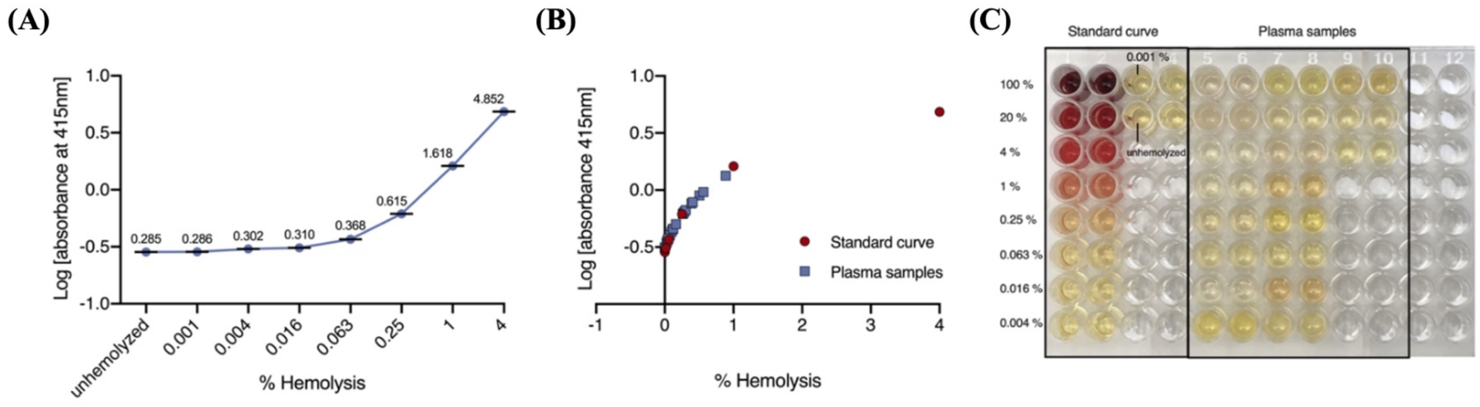
(A)
Log [absorbance at 415nm]
1.0
0.5
0.0
-0.5
-1.0
0.285
0.286
0.302
0.310
0.368
0.615
1.618
4.852
unhemolyzed
0.001
0.004
0.016
0.063
0.25
1
4
% Hemolysis
(B)
Log [absorbance 415nm]
1.0
0.5
0.0
-0.5
-1
0
1
2
3
4
Standard curve
Plasma samples
% Hemolysis
(C)
Standard curve
Plasma samples
0.001 %
unhemolyzed
100 %
20 %
4 %
1 %
0.25 %
0.063 %
0.016 %
0.004 %

Figure S2

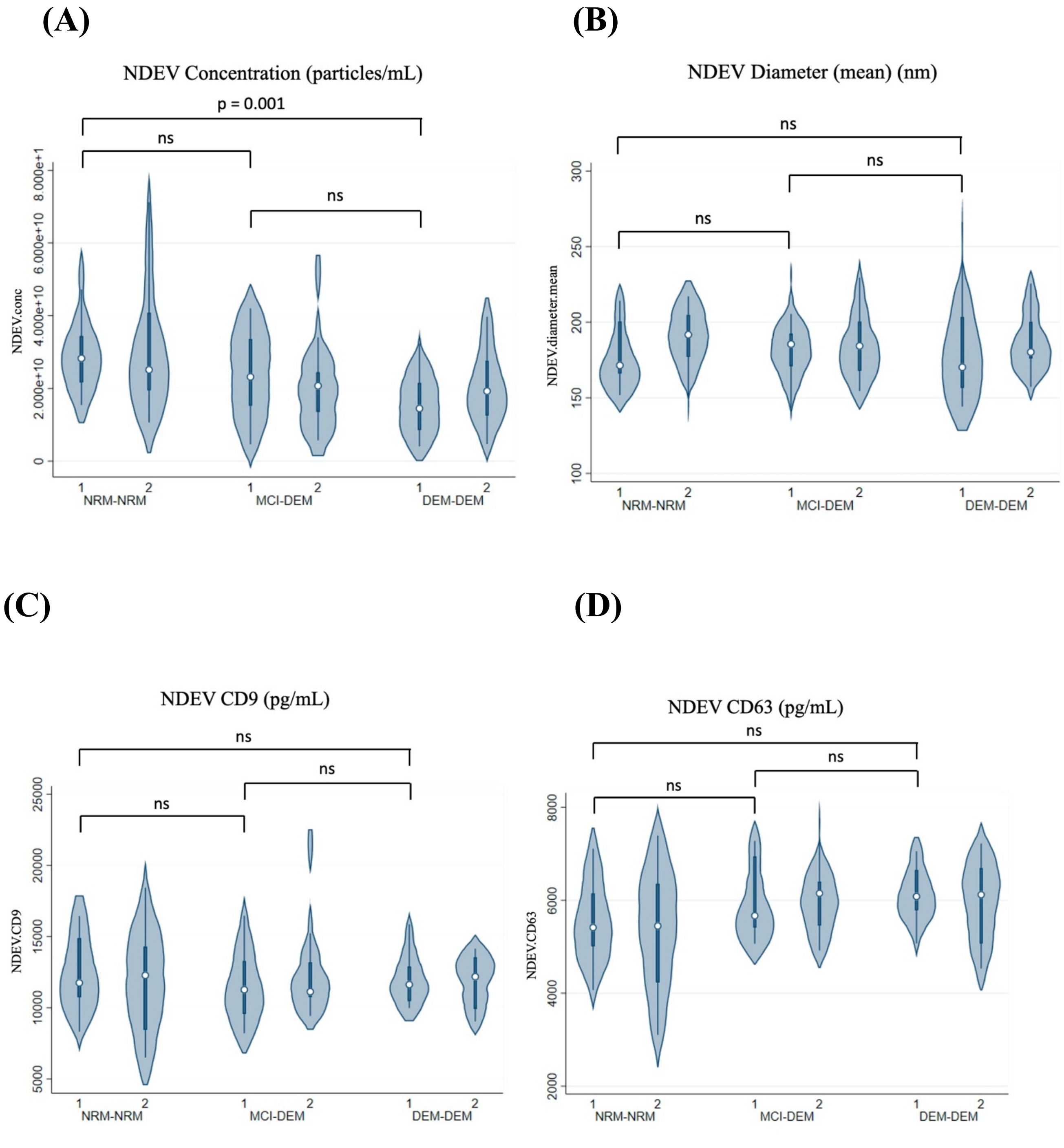
(A)
NDEV Concentration (particles/mL)
p = 0.001
ns
ns
NDEV.conc
0
2.000e+10
4.000e+10
6.000e+10
8.000e+1
1
2
NRM-NRM
MCI-DEM
DEM-DEM
(B)
NDEV Diameter (mean) (nm)
ns
ns
ns
NDEV.diameter.mean
100
150
200
250
300
1
2
NRM-NRM
MCI-DEM
DEM-DEM
(C)
NDEV CD9 (pg/mL)
ns
ns
ns
NDEV.CD9
5000
10000
15000
20000
25000
1
2
NRM-NRM
MCI-DEM
DEM-DEM
(D)
NDEV CD63 (pg/mL)
ns
ns
ns
NDEV.CD63
2000
4000
6000
8000
1
2
NRM-NRM
MCI-DEM
DEM-DEM

(E)

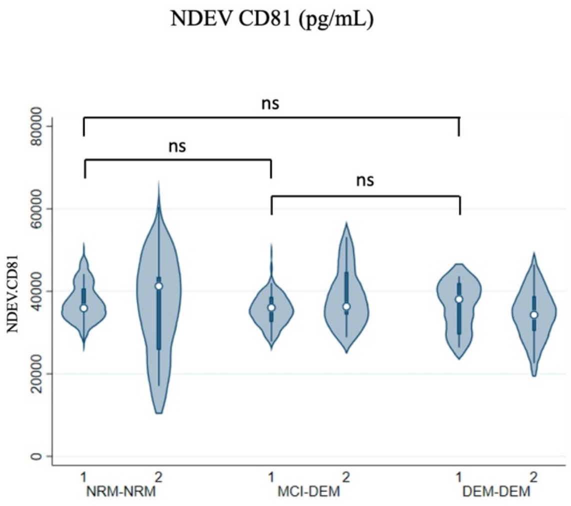
NDEV CD81 (pg/mL)
ns
ns
ns
NDEV.CD81
0
20000
40000
60000
80000
1
2
NRM-NRM
1
2
MCI-DEM
1
2
DEM-DEM

(F)

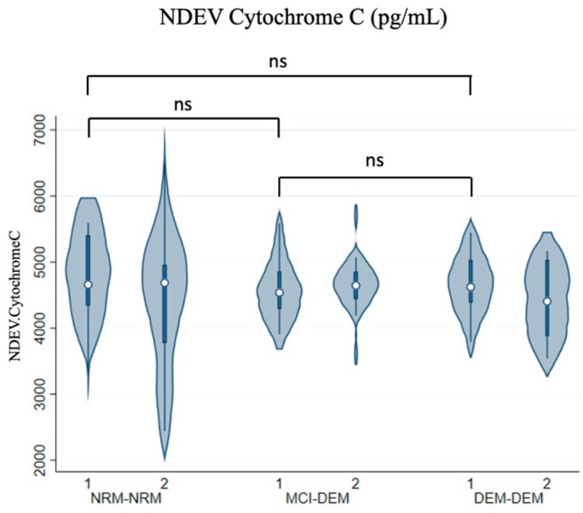
NDEV Cytochrome C (pg/mL)
ns
ns
ns
NDEV.CytochromeC
2000
3000
4000
5000
6000
7000
1
2
NRM-NRM
1
2
MCI-DEM
1
2
DEM-DEM

(G)

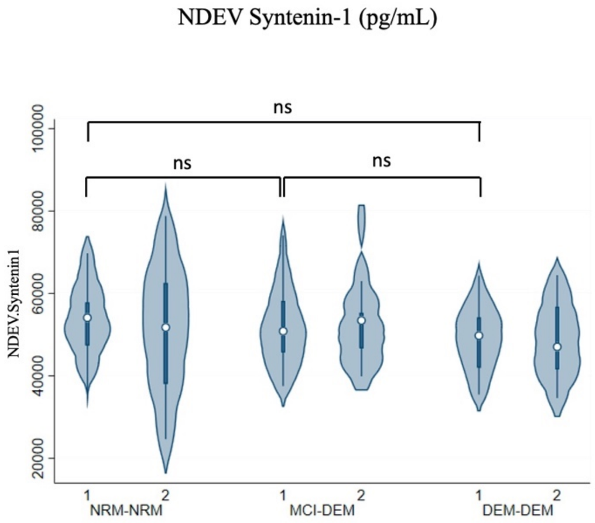
NDEV Syntenin-1 (pg/mL)
ns
ns
ns
NDEV.Syntenin1
20000
40000
60000
80000
100000
1
2
NRM-NRM
1
2
MCI-DEM
1
2
DEM-DEM

(H)

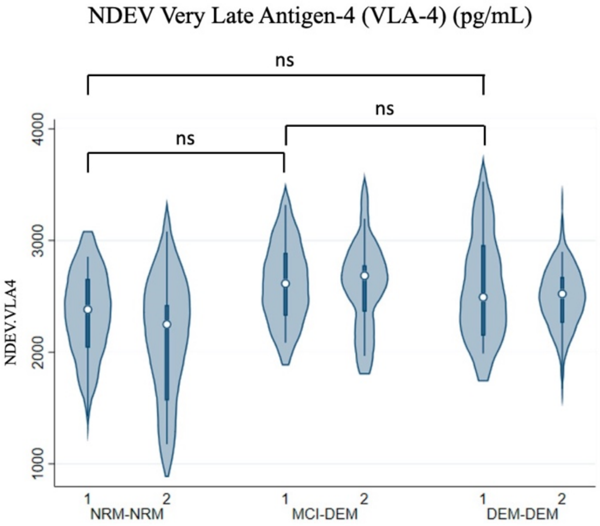
NDEV Very Late Antigen-4 (VLA-4) (pg/mL)
ns
ns
ns
NDEV.VLA4
1000
2000
3000
4000
1
2
NRM-NRM
1
2
MCI-DEM
1
2
DEM-DEM

Figure S3

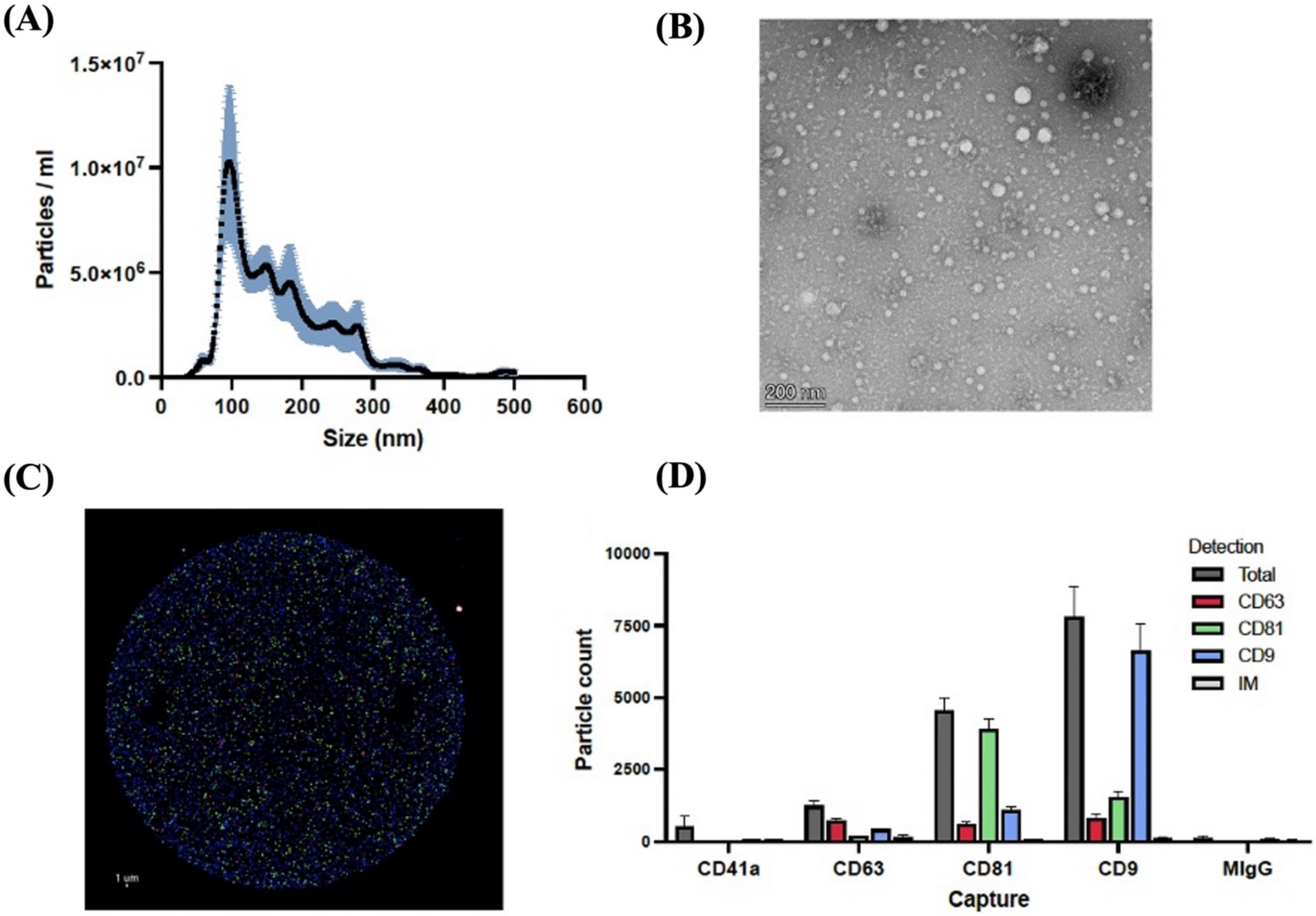

(A)
1.5×10^7
1.0×10^7
5.0×10^6
0.0
Particles / ml
0
100
200
300
400
500
600
Size (nm)
(B)
200 nm
(C)
1 um
(D)
Detection
Total
CD63
CD81
CD9
IM
10000
7500
5000
2500
0
Particle count
CD41a
CD63
CD81
CD9
MIgG
Capture

Figure S4

(A)

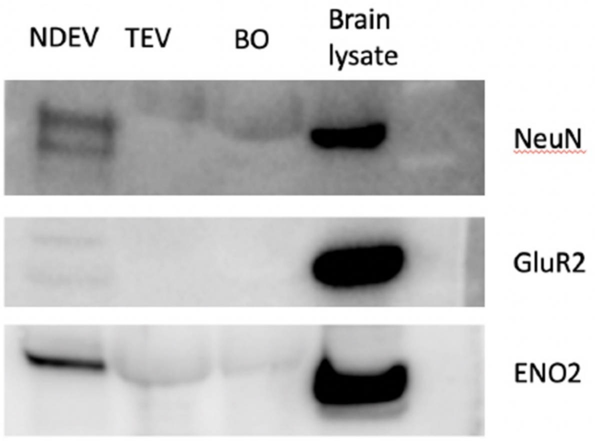
NDEV
TEV
BO
Brain lysate
NeuN
GluR2
ENO2

(B)

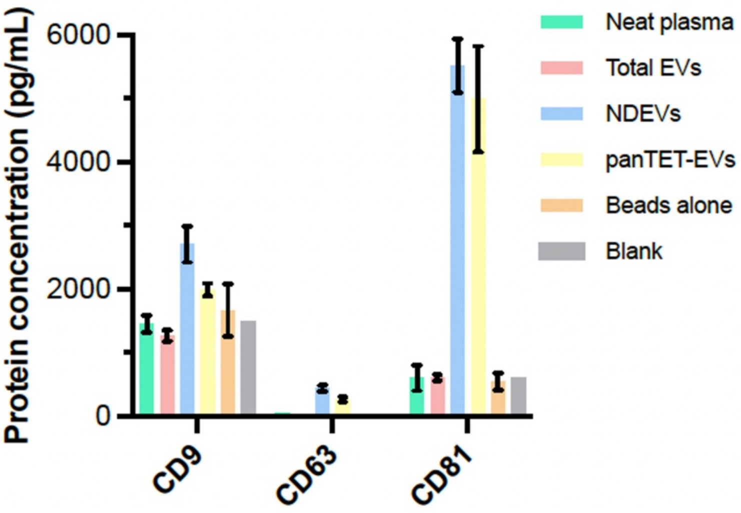
Protein concentration (pg/mL)
6000
4000
2000
0
CD9
CD63
CD81
Neat plasma
Total EVs
NDEVs
panTET-EVs
Beads alone
Blank

Figure S5

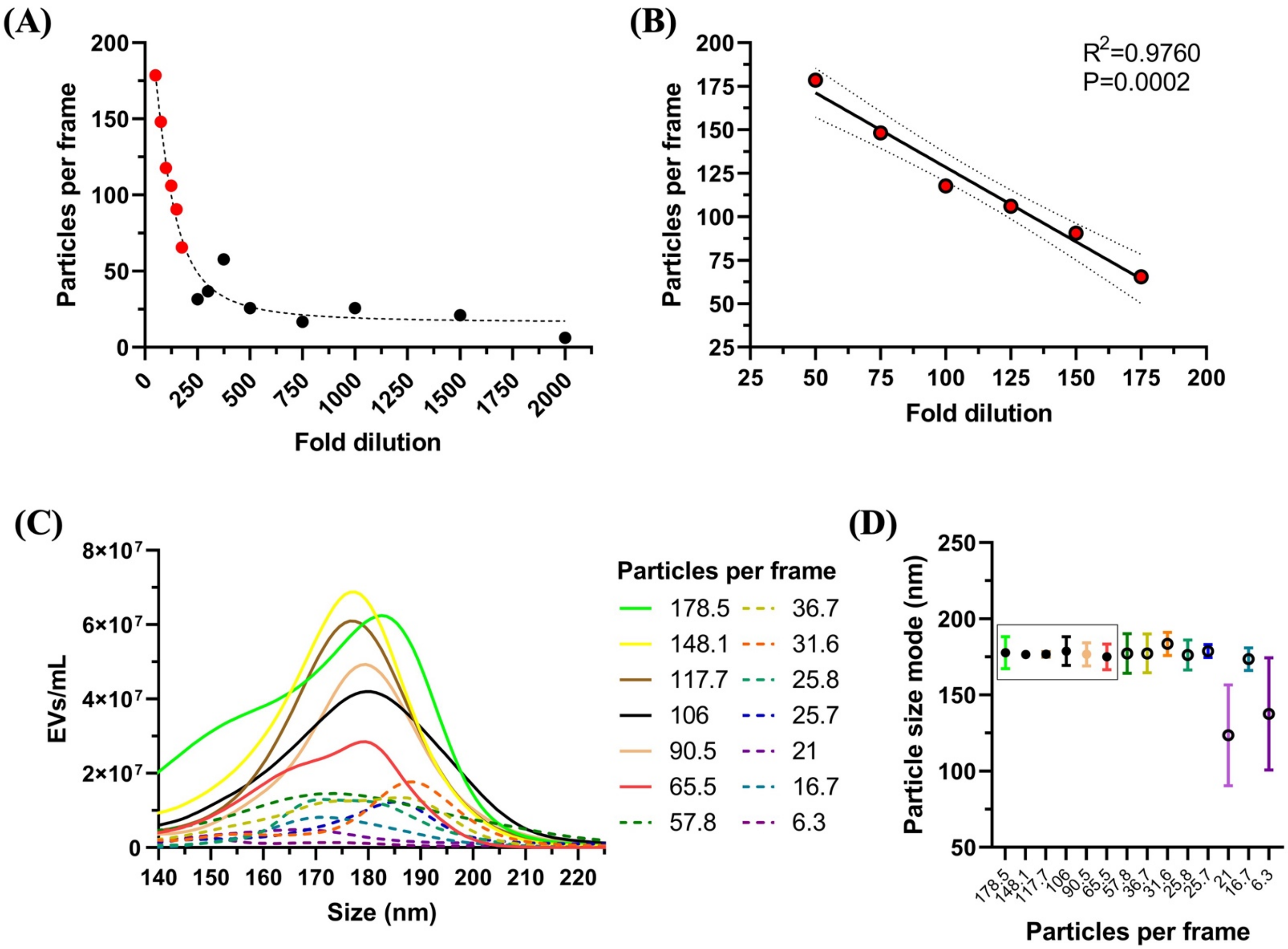
(A)
Particles per frame
Fold dilution
(B)
R2=0.9760
P=0.0002
Particles per frame
Fold dilution
(C)
EVs/mL
Size (nm)
Particles per frame
178.5
148.1
117.7
106
90.5
65.5
57.8
36.7
31.6
25.8
25.7
21
16.7
6.3
(D)
Particle size mode (nm)
Particles per frame

Figure S6

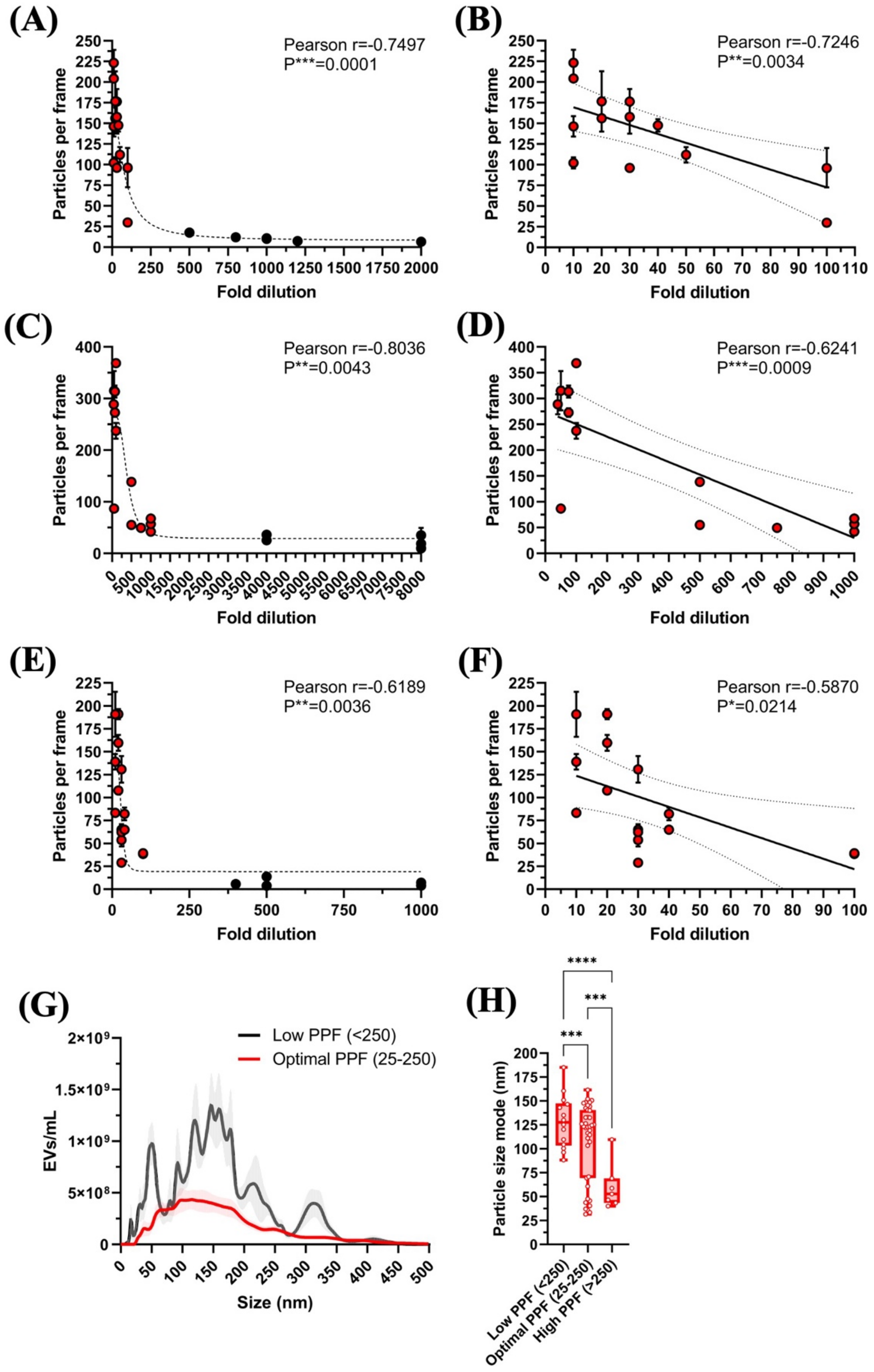

(A)
Pearson r=-0.7497
P***=0.0001
Particles per frame
Fold dilution
(B)
Pearson r=-0.7246
P**=0.0034
Particles per frame
Fold dilution
(C)
Pearson r=-0.8036
P**=0.0043
Particles per frame
Fold dilution
(D)
Pearson r=-0.6241
P***=0.0009
Particles per frame
Fold dilution
(E)
Pearson r=-0.6189
P**=0.0036
Particles per frame
Fold dilution
(F)
Pearson r=-0.5870
P*=0.0214
Particles per frame
Fold dilution
(G)
Low PPF (<250)
Optimal PPF (25-250)
EVs/mL
Size (nm)
(H)
****
***
***
Particle size mode (nm)
Low PPF (<250)
Optimal PPF (25-250)
High PPF (>250)

Figure S7

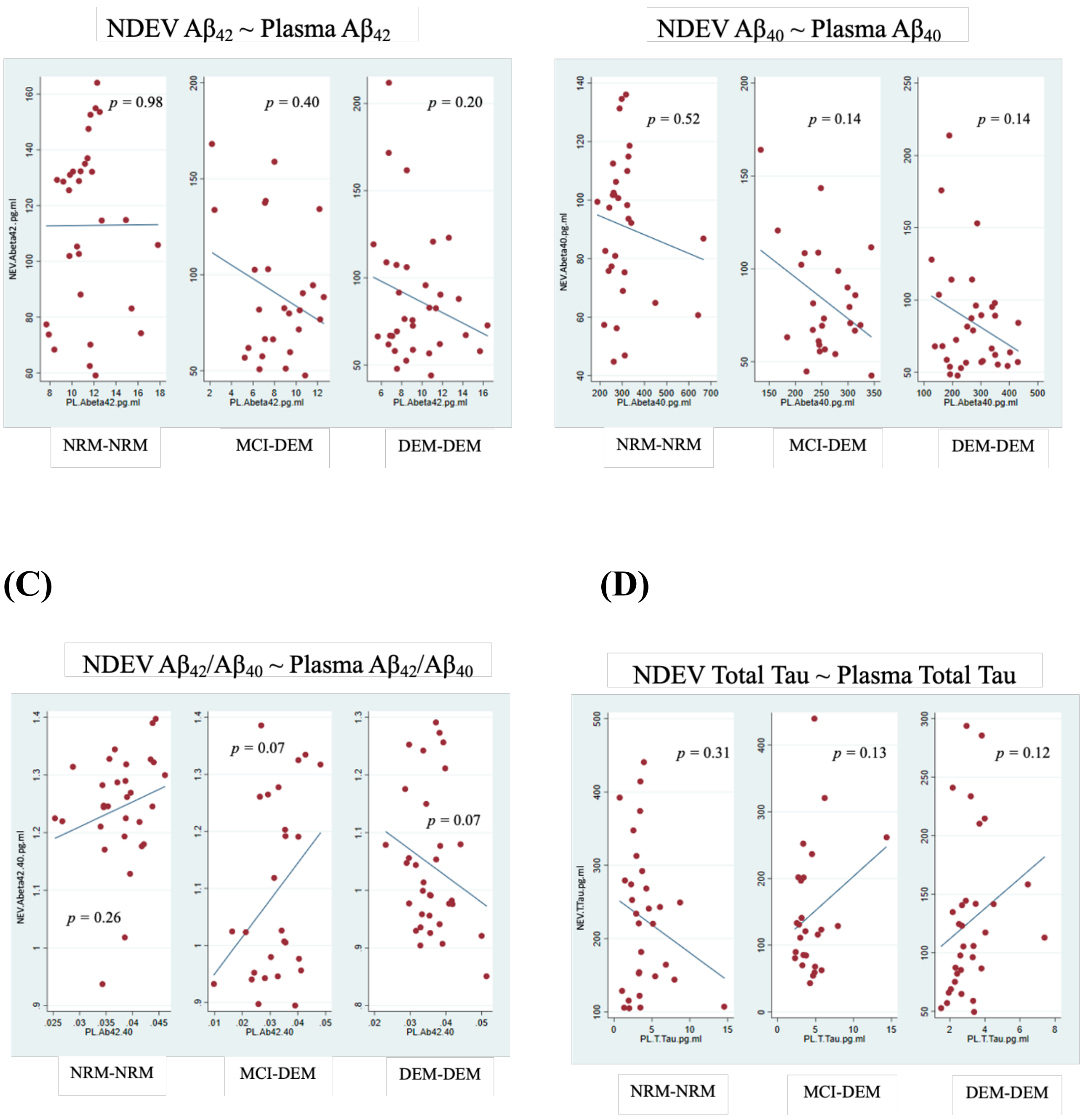

(A)
NDEV Aβ_{42} ~ Plasma Aβ_{42}
NEV.Abeta42.pg.ml
PL.Abeta42.pg.ml
p = 0.98
p = 0.40
p = 0.20
NRM-NRM
MCI-DEM
DEM-DEM
(B)
NDEV Aβ_{40} ~ Plasma Aβ_{40}
NEV.Abeta40.pg.ml
PL.Abeta40.pg.ml
p = 0.52
p = 0.14
p = 0.14
NRM-NRM
MCI-DEM
DEM-DEM
(C)
NDEV Aβ_{42}/Aβ_{40} ~ Plasma Aβ_{42}/Aβ_{40}
NEV.Abeta42.40.pg.ml
PL.Ab42.40
p = 0.26
p = 0.07
p = 0.07
NRM-NRM
MCI-DEM
DEM-DEM
(D)
NDEV Total Tau ~ Plasma Total Tau
NEV.TTau.pg.ml
PL.T.Tau.pg.ml
p = 0.31
p = 0.13
p = 0.12
NRM-NRM
MCI-DEM
DEM-DEM

(E)

NDEV P181-Tau ~ Plasma P181-Tau

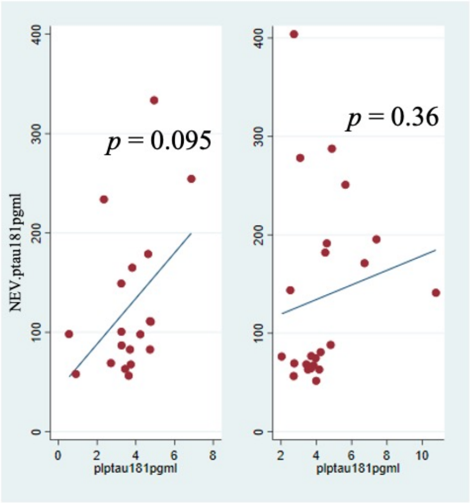

MCI-DEM DEM-DEM

Table S1. Summary of non-Box-Cox transformed marker measurements

Variable	NRM-NRM			MCI-DEM			DEM-DEM		
	Visit 1	Visit 2	Change per year	Visit 1	Visit 2	Change per year	Visit 1	Visit 2	Change per yar
**NDEV MEASURES**									
**NDEV Concentration (particles/mL)**	2.99e+10 (1.09e+10)	3.18e+10 (1.75e+10)	-4.27e+08 (7.41e+09)	2.38e+10 (1.25e+10)	2.06e+10 (1.27e+10)	-1.09e+09 (5.05e+09)	1.57e+10 (7.94e+09)	2.10e+10 (1.02e+10)	2.96e+09 (6.43e+09)
**NDEV Diameter (mean) (nm)**	178.114 (22.212)	189.107 (19.807)	4.662 (13.245)	183.285 (22.416)	186.6 (23.493)	3.474 (11.436)	181.212 (31.535)	187.294 (19.668)	1.55 (11.996)
**NDEV Diameter (mode) (nm)**	132.386 (16.945)	139.171 (17.216)	1.987 (6.413)	138.546 (19.427)	156.062 (38.94)	12.112 (26.273)	139.659 (14.978)	153.125 (21.526)	8.046 (12.371)
**CD9 (pg/mL)**	12465.57 (2645.534)	11994.06 (3600.272)	-24.955 (1419.676)	11657.32 (2598.353)	12455.38 (3155.826)	101.009 (1037.748)	12070.59 (1766.872)	11723.46 (1879.586)	-203.449 (1603.734)
**CD63 (pg/mL)**	5554.842 (896.401)	5342.217 (1272.762)	-67.702 (487.993)	6013.343 (826.668)	6020.135 (746.776)	-59.852 (566.58)	6105.306 (585.478)	5919.857 (900.846)	-28.949 (686.254)
**CD81 (pg/mL)**	37681.74 (5914.339)	36798.27 (12535.11)	-122.268 (5362.876)	36413.56 (5689.352)	39014.64 (7334.546)	141.455 (1729.388)	36138.07 (5894.765)	34368.46 (6691.483)	-2164.725 (4919.348)
**Cytochrome C (pg/mL)**	4746.052 (701.067)	4447.543 (1127.067)	-94.015 (472.751)	4594.068 (504.27)	4652.342 (503.709)	31.715 (335.729)	4636.875 (473.456)	4408.457 (562.705)	-119.182 (370.45)
**Syntenin-1 (pg/mL)**	53713.27 (9425.329)	51494.63 (15521.98)	-697.349 (7667.574)	52556.04 (10468.91)	52857.9 (10503.76)	-2224.182 (5581.718)	49034.35 (7915.8)	48641.09 (8921.617)	-105.412 (6927.42)
**VLA-4 (pg/mL)**	2302.961 (419.884)	2137.981 (554.862)	-61.997 (281.134)	2631.273 (372.165)	2615.838 (444.751)	-30.668 (206.399)	2605.081 (469.995)	2494.399 (394.309)	-100.488 (473.08)
**Aβ_{42} (pg/mL)**	115.14 (24.04)	106.882 (36.049)	-2.81 (12.884)	83.785 (35.466)	96.39 (34.391)	9.947 (20.563)	92.37 (48.327)	82.743 (21.452)	-10.324 (25.751)
**Aβ_{40} (pg/mL)**	93.082 (19.067)	85.745 (28.937)	-2.351 (11.373)	77.005 (32.152)	88.225 (27.706)	8.512 (17.553)	89.132 (48.679)	80.55 (22.657)	-9.282 (23.048)

**$A\beta_{42}/A\beta_{40}$**	1.24 (0.109)	1.249 (0.095)	0 (0.025)	1.093 (0.162)	1.089 (0.164)	-0.001 (0.033)	1.046 (0.121)	1.04 (0.129)	-0.009 (0.035)
**Total Tau (pg/mL)**	225.375 (87.804)	214.49 (106.756)	-4.479 (35.067)	130.038 (71.973)	163.317 (112.991)	22.101 (48.5)	130.355 (80.301)	125.652 (49.509)	-13.8 (47.553)
**P181-Tau (pg/mL)**	197.96 (71.32)	185.377 (86.95)	-3.157 (41.587)	131.102 (81.723)	136.24 (67.513)	10.135 (35.276)	142.262 (99.6)	126.779 (66.554)	-22.561 (67.808)
**P231-Tau (pg/mL)**	418.381 (94.283)	370.011 (84.994)	-30.962 (72.481)	425.168 (122.893)	409.531 (70.9)	-9.132 (44.16)	416.817 (87.512)	372.351 (89.316)	-26.724 (53.67)
**proBDNF (pg/mL)**	14.585 (2.977)	13.109 (3.012)	-0.491 (1.344)	9.74 (3.848)	10.551 (3.707)	0.57 (1.411)	9.794 (4.726)	9.079 (3.419)	-0.946 (2.833)
**BDNF (pg/mL)**	30.431 (7.399)	32.499 (8.458)	-0.454 (4.811)	27.074 (4.986)	30.274 (6.92)	0.988 (4.196)	29.908 (7.137)	32.245 (5.926)	0.342 (4.405)
**NRGN (pg/mL)**	340.812 (57.107)	324.625 (56.961)	-11.685 (36.948)	325.524 (60.907)	337.053 (65.377)	8.357 (42.769)	329.76 (105.285)	307.912 (69.566)	-27.901 (81.403)
**a-syn (pg/mL)**	278.339 (26.174)	275.43 (22.944)	-4.198 (10.528)	290.245 (26.81)	299.713 (31.919)	7.167 (20.372)	293.999 (44.035)	290.385 (38.201)	-7.383 (30.707)
**NfH (pg/mL)**	572.806 (94.516)	569.034 (114.444)	-12.128 (63.076)	603.563 (81.551)	631.731 (93.863)	11.432 (56.364)	608.234 (151.032)	596.099 (104.932)	-20.6 (107.158)
**TDP-43 (pg/mL)**	6747.372 (1552.984)	6497.803 (1743.796)	-272.345 (961.764)	7179.178 (1570.361)	7046.781 (1681.157)	-143.692 (706.17)	6995.157 (1481.272)	6625.506 (1453.816)	-298.063 (1080.933)
**PLASMA MEASURES**									
**$A\beta_{42}$ (pg/mL)**	10.912 (2.422)	11.542 (2.24)	0.162 (0.763)	8.09 (2.85)	8.339 (2.753)	0.076 (1.094)	9.766 (2.759)	9.079 (2.995)	-0.137 (0.891)
**$A\beta_{40}$ (pg/mL)**	300.52 (109.713)	319.452 (111.106)	5.325 (27.276)	250.96 (54.722)	263.517 (51.434)	2.058 (40.299)	274.16 (72.264)	263.51 (101.981)	-1.301 (45.167)
**$A\beta_{42}/A\beta_{40}$**	0.037 (0.005)	0.037 (0.005)	0 (0.002)	0.032 (0.008)	0.032 (0.01)	0.001 (0.003)	0.036 (0.006)	0.036 (0.006)	0 (0.003)
**Total Tau (pg/mL)**	3.975 (2.366)	3.993 (3.332)	0.11 (1.275)	4.682 (3.089)	4.283 (1.645)	-0.161 (1.147)	3.019 (0.773)	3.304 (1.611)	-0.018 (0.59)
**P181-Tau (pg/mL)**	3.475 (1.153)	0.025		3.432 (1.528)	3.911 (1.375)	-0.116 (0.505)	4.468 (2.284)	4.168 (1.351)	0.19 (0.476)

Values presented are mean (standard error).

Reference

HARBOE, M. 1959. A method for determination of hemoglobin in plasma by near-ultraviolet spectrophotometry. *Scand J Clin Lab Invest,* 11**,** 66-70.
